# Molecular Processes Leading to Shear Banding in Entangled Polymeric Solutions

**DOI:** 10.3390/polym15153264

**Published:** 2023-07-31

**Authors:** Mahdi Boudaghi, Brian J. Edwards, Bamin Khomami

**Affiliations:** Materials Research and Innovation Laboratory, Chemical and Biomolecular Engineering, University of Tennessee, Knoxville, TN 37996, USA; mboudagh@vols.utk.edu

**Keywords:** rheology, complex fluids, shear banding, nonlinear dynamics, flow instability, dissipative particle dynamics, nonequilibrium molecular dynamics

## Abstract

The temporal and spatial evolution of shear banding during startup and steady-state shear flow was studied for solutions of entangled, linear, monodisperse polyethylene $C_{3000}H_{6002}$ dissolved in hexadecane and benzene solvents. A high-fidelity coarse-grained dissipative particle dynamics method was developed and evaluated based on previous NEMD simulations of similar solutions. The polymeric contribution to shear stress exhibited a monotonically increasing flow curve with a broad stress plateau at intermediate shear rates. For startup shear flow, transient shear banding was observed at applied shear rates within the steady-state shear stress plateau. Shear bands were generated at strain values where the first normal stress difference exhibited a maximum, with lifetimes persisting for up to several hundred strain units. During the lifetime of the shear bands, an inhomogeneous concentration distribution was evident within the system, with higher polymer concentration in the slow bands at low effective shear rate; i.e., $\dot{\gamma }<\tau_{R}^{-1}$, and vice versa at high shear rate. At low values of applied shear rate, a reverse flow phenomenon was observed in the hexadecane solution, which resulted from elastic recoil of the molecules within the slow band. In all cases, the shear bands dissipated at high strains and the system attained steady-state behavior, with a uniform, linear velocity profile across the simulation cell and a homogeneous concentration.

## 1. Introduction

Shear banding is defined as the formation of localized zones with different shear rates (and hence different velocity profiles) in a classical steady or transient shear flow. It has become established as a common phenomenon observed in the flow of soft matter, such as entangled polymeric melts and solutions. However, the impact of shear banding on many polymer processing instabilities, such as distortions in extruded plastics like the sharkskin and surface fracture [1,2], remain incompletely understood. To that end, this phenomenon has been studied extensively using theory, computation, and experiments. These investigations have led to significant advances in the understanding of the underlying physics that leads to shear banding in polymer solutions and melts, and recent insights will inevitably lead to the development of first principle models and design tools in polymer processing applications.

Reptation-based studies of this phenomenon [3,4] attribute shear banding in simple shear flow to a mechanical instability that arises solely due to a nonmonotonic steady-state shear stress versus strain rate flow profile. However, numerous studies have demonstrated that steady and transient shear banding occurs in the shear flow of entangled polymeric fluids with a monotonic flow curve, i.e., $\partial \sigma_{xy}/\partial \dot{\gamma }\geq 0$, where $\sigma_{xy}$ is steady-state shear stress and $\dot{\gamma }$ is the shear rate [5,6,7,8,9,10]. Hence, a nonmontonic flow curve is not a prerequisite for formation of banded structures. In fact, coarse-grained atomistic simulations of entangled polymeric melts have shown that this phenomenon occurs as a result of heterogeneous disentanglement in the gradient direction that results from spatial variation in segmental orientation and stretching. This reduction in the entanglement network culminates in a commensurate rotation of the macromolecules in the flow-gradient plane after the overshoot in the normal stress commonly observed in step-strain startup of shear flow at Wi≥1. The local variation in the macromolecular configurations in turn gives rise to localized velocity perturbations, which can ultimately result in alternating layers of high and low local shear rates [11,12,13,14,15,16]. Specifically, Mohaghegi and Khomami [13] and Boudaghi et al. [11,12] have determined that in startup step-strain flows, onset of shear banding corresponds to shear rates of order $\dot{\gamma }\approx O\left(\tau_{R}^{-1}\right)$, where $\tau_{R}$ is the characteristic Rouse time that governs the polymer segmental stretching. Specifically, steady shear banding has been shown to occur at $\dot{\gamma }\leq O\left(\tau_{R}^{-1}\right)$, while transient shear banding has been observed at $\dot{\gamma }>O\left(\tau_{R}^{-1}\right)$. Although much progress has been made toward mechanistic understanding of shear banding in entangled polymeric melts, a detailed understanding of this phenomenon, particularly in entangled polymeric solutions, remains an area of active research.

It is well known that the coupling of concentration fluctuations and flow dynamics in polymeric solutions can lead to shear-induced migration of macromolecules. In the slow shear flow limit, the phenomenological theory of Helfand and Fredrickson (HF) attributes shear-induced migration to the coupling between flow-enhanced concentration fluctuations and the concentration dependence of the viscosity and normal stress coefficients [17]. The seminal HF theory has served as a starting point for more detailed models of shear-induced migration of macromolecules [18,19,20,21]. Overall, these models correctly capture the anisotropic enhancement of concentration fluctuations and shear-induced phase transitions in flowing polymer solutions as functions of strain rate and temperature [22]. Furthermore, the butterfly-shaped scattering patterns measured via small-angle light scattering and small-angle neutron scattering of flowing concentrated solutions clearly demonstrate that shear-induced fluctuations [23,24,25] give rise to concentration inhomogeneity and a commensurate concentration dependence of the viscosity and normal stress coefficients. Furthermore, coupling between flow-induced concentration fluctuations and the concentration dependence of the viscometric functions of polymeric solutions can manifest as shear-induced migration of the macromolecules [17,23,26,27,28,29].

In recent years, the interplay between concentration fluctuations, shear and normal stresses, and the ensuing flow-induced migration of the polymer chains has been identified as a prerequisite to formation of shear-banded flows of entangled polymeric solutions. To that end, theoretical analysis based on the two-fluid approach (i.e., solvent and polymer as interpenetrating liquids) has been used to examine the role of shear-induced macromolecular migration on the formation of banded velocity profiles. Specifically, Fielding and Olmsted [30] used a two-fluid approach with a modified nonlocal Johnson–Segalman model [31,32] and a nonmonotonic flow curve to develop a phase diagram that delineated the role of shear-induced migration on shear banding. This analysis demonstrated that for steady shear-banded flow, the high shear-rate region of shear-banded solutions might have a lower concentration. Later, the Rolie–Poly constitutive model [33] with a coupled stress-concentration two-fluid approach was used by Cromer et al. [34] to investigate shear banding of polymeric solutions with a monotonic flow curve. Their results indicated a flow-induced concentration redistribution and a shear-banded flow structure with a lower concentration within the high shear band (adjacent to the moving wall). Moreover, a jump in the polymer contribution to the total stresses of the high and low shear-rate bands was reported. Evidently, this jump is a consequence of using a continuum-level constitutive equation for the description of polymer dynamics.

Most recently, Burroughs et al. have provided experimental evidence of the coupling between flow-induced concentration nonuniformity in a shear-banded flow [35,36]. Specifically, experiments in a Taylor–Couette flow geometry were performed using in situ rheo-particle tracking velocimetry to obtain the velocity and concentration profiles for a polybutadiene/dioctyl phthalate solution with an average entanglement number of 38. Additionally, analysis was performed with a two-fluid model with the Rolie–Poly constitutive equation. Both experiments and theoretical analysis predicted a lower polymer concentration in the fast band and a higher concentration in the slow band under steady-state conditions. Based on these observations, it was postulated that a nonuniform polymer concentration in the gradient direction was a prerequisite for incipient shear banding. Furthermore, the authors suggested that their observed wall slip and/or wall depletion layer did not greatly influence the underlying mechanism that gave rise to shear banding in these entangled polymer solutions.

Although these studies provide evidence that shear-induced migration can cause shear banding, a number of issues must be resolved before one can unequivocally claim that shear banding in entangled polymeric solutions is a consequence of shear-induced migration of macromolecules. For instance, the time scale for development of concentration fluctuations is extremely long (≈$10^{6}\tau_{d})$, where $\tau_{d}$ is the disengagement time. Furthermore, the influence of curvature and associated hoop stresses on formation of a banded flow structure requires clarification. Last but not least, the results of these studies are at odds with high-resolution planar large-amplitude oscillatory flow confocal microscopy/rheometry of a polymeric solution composed of λ-DNA with an average entanglement number of 300 [37]. These experiments demonstrated that shear-banded velocity profiles occur at high flow rates (Wi≫1) at significantly shorter time scales without any evidence of a concentration difference between the high and low shear bands. Moreover, these experimental observations support earlier coarse-grained molecular dynamics simulation results [11,13,14,15] that attributed incipient shear banding to an inhomogeneous entanglement density in the flow-gradient plane.

To further elucidate the mechanistic aspects of shear banding, high-fidelity coarse-grained dissipative particle dynamics (DPD) simulations of entangled polyethylene solutions were performed over a wide range of shear rates using both n-hexadecane and benzene as solvents. The adopted simulation strategy allows one to consider the effects of temperature, molecular weight, and solvent quality on flow-induced concentration fluctuations and shear-induced concentration gradients that have been neglected in much of the prior theoretical and experimental studies of shear banding in entangled polymeric solutions. The DPD model and simulation methodology are described and validated in Section 2. Results of the simulations are presented in Section 3, and the conclusions from the simulation study are summarized in Section 4.

## 2. DPD Model Development and Validation

The dissipative particle dynamics force-field model [38,39,40,41] was used to perform high-fidelity large-scale simulations of entangled polymer solutions of linear monodisperse polyethylene (PE) $C_{3000}H_{6002}$, corresponding to a PE of molar mass 42,002 g/mol, dissolved in oligomeric n-hexadecane and aromatic benzene solvents. An accurate prediction of polymer dynamics in solutions requires the correct parameterization of the course-grained DPD force field in order to capture the intramolecular and intermolecular interactions between both polymer and solvent particles. To this end, a recently developed method [42] was used to parameterize and fine-tune the DPD model through the direct mapping of data from united-atom NEMD simulations to the DPD representation. Hence, the two solvents studied herein were chosen to match those used in the NEMD simulations so that a direct mapping of the DPD system to its atomistic analog could be achieved.

Three types of nonbonded pairwise forces were incorporated in the model: a repulsive conservative force, $F^{C}$, a dissipative force, $F^{D}$, and a random (Brownian) force, $F^{R}$, acting on each particle. The forces within the DPD cutoff distance, $r_{c_{a}}$, vary with the distance between paired particle centers, $r_{ij}$, and act in a direction of unit vector ${\hat{e}}_{ij}\left(=r_{ij}/r_{ij}\right)$, i.e., the shortest distance between the particles. The DPD forces on each particle *i* are described by the equations

(1) $$F_{i}^{DPD}=\sum_{j\neq i}\left(F_{ij}^{C}+F_{ij}^{D}+F_{ij}^{R}\right)\;,$$

(2) $$F_{ij}^{C}=a\left(1-\frac{r_{ij}}{r_{c_{a}}}\right){\hat{e}}_{ij}\;,$$

(3) $$F_{ij}^{D}=-\gamma^{D}\omega^{D}r_{ij}\left[\left(v_{i}-v_{j}\right).{\hat{e}}_{ij}\right]{\hat{e}}_{ij}\;,$$

(4) $$F_{ij}^{R}=\sigma \omega^{R}r_{ij}\zeta {\hat{e}}_{ij}\;,$$

where

(5) $$\omega^{D}={\left(\omega^{R}\right)}^{2}={\left(1-r_{ij}/r_{c_{a}}\right)}^{2}\;.$$

The conservative force $F^{C}$ is repulsive and decreases linearly from its maximum value at $r_{ij}=0$ of $a_{ij}$, where $a_{ij}$ is the conservative force parameter between particle *i* and particle *j*. $F^{D}$ and $F^{R}$ ensure the correct hydrodynamic interaction between the particles by linking the velocity differences between all pairs of particles, $v_{i}-v_{j}$, and the temperature of the system, respectively. Specifically, the fluctuation–dissipation theorem establishes a connection between the viscous dissipation and system temperature as $\sigma^{2}=2\gamma^{D}k_{B}T$, where $\gamma^{D}$, σ, and $k_{B}T$ are the viscous friction coefficient, the magnitude of thermal noise, and the effective system temperature, respectively. Furthermore, the forces are coupled through two weighting functions for the dissipative, $\omega^{D}$, and random forces, $\omega^{R}$, by Equation (5). To avoid temporal divergence caused by large temperature fluctuations, the coefficient of viscous friction was set at $\gamma^{D}=4.5$ [41]. The unitless simulation temperature was set to unity ($k_{B}T=1$) for all simulations in this study. Consequently, the DPD time scale unit was defined as $\tau_{DPD}={\left(mr_{c_{a}}^{2}/k_{B}T\right)}^{0.5}$. In prior work [43], based on atomistic simulations of PE solutions of $C_{1000}H_{2002}$ in hexadecane and benzene, simulation temperatures corresponded to real units of 391 K and 353 K, respectively; the simulated temperatures of the $C_{3000}H_{6002}$ solutions are deemed to be similar.

In addition to nonbonded interactions, polyethylene (and n-hexadecane) chains were formed by connecting each adjacent DPD particle along the chain backbone with a harmonic bond force, $F_{ik}^{spr}=-k_{spr}\left(b_{eq}-b_{ik}\right)$, for k=i±1, where $k_{spr}$, $b_{eq}=0.95r_{c_{a}}$, and $b_{ik}$ are the spring force constant, equilibrium average bond length, and instantaneous bond length between adjacent particles. Additionally, to obtain an optimal mapping between atomistic polyethylene chains and DPD model PE chains, a bending potential force was introduced, $F_{il}^{bend}=-k_{bend}\left(1-\sin\left(\theta \right)\right)$, for l=i±2, where $k_{bend}$ and θ are the force constant and the angle between three successive particles along the chain backbone [42]. The sum of all bonded and nonbonded interactions results in the total force acting on each particle as

(6) $$F_{i}^{tot}=F_{i}^{DPD}+F_{i}^{spr}+F_{i}^{bend}\;.$$

Molecular simulations of entangled polymers require preventative measures to avoid bond crossing. A simple topological constraint developed by Nikunen et al. [44]; i.e., $\sqrt{2}r_{min}>l_{max}$ where $r_{min}$(=0.77) is the effective radius of the particles and $l_{max}$(=1.05) is the maximum distance between adjacent beads in the backbone, was employed to avoid aphysical bond crossing. Note that these parameters were calculated based on the pair correlation function, g(r), and probability density function of bond length, $P(r_{eq})$. The DPD tuning parameters have been discussed in previous work [12,42,44,45]. A more detailed description of the DPD model and force field can be found in prior publications [11,42,45,46].

**Figure 1:**
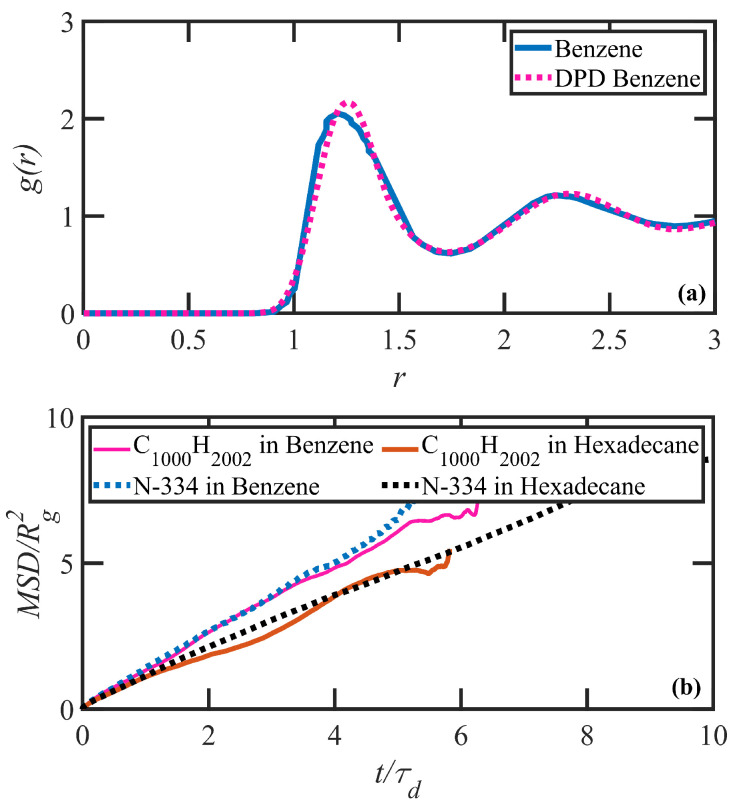
(**a**) The pair correlation functions, g(r), for a benzene liquid obtained via experiment [48,49] (solid blue curve) and DPD simulation (dotted red curve). Note that the experimental data have been rescaled to DPD units. (**b**) The mean-squared displacement of the center of mass of the N-334 DPD and $C_{1000}H_{2002}$ PE chains dissolved in n-hexadecane and benzene. The data from molecular dynamics simulations are presented with solid lines, and the data from the DPD simulations are displayed using dotted lines.

The recent comparisons of NEMD and DPD simulations of linear polyethylene melts [42] have demonstrated the efficacy of lumping three $CH_{2}$ monomers into one DPD particle, with adjacent particles being connected through the harmonic spring force field. Hence, the DPD chains corresponding to $C_{3000}H_{6002}$ macromolecules are composed of 1000 DPD particles, which is referred to as PE N-1000 herein. The mass of each alkane DPD particle was set to unity, $m_{a}=1$, as was the force cut-off distance, $r_{c_{a}}=1$, and a reduced particle density of $\rho_{a}=1$ was assumed. The n-hexadecane ($C_{16}H_{34}$) solvent molecules were formed using 6 DPD particles. To this end, the $k_{bend}$ parameter was reduced from 2.38 to 2.0 to optimize the mapping parameter simultaneously while decreasing the methylene group to DPD ratio from 3:1 to approximately 2.8:1 (please see Ref. [42]). Other simulation coefficients were adopted similarly to the N-1000 system. Note that the effect of the bending potential on the level of coarse graining was discussed in detail in previous work [42]. The benzene ($C_{6}H_{6}$) solvent molecules were modeled as a single DPD particle. The unitless molar mass and interaction cut-off distance for benzene particles were assigned based on the molecular weight and molar volume using the Durchschlag and Zipper rule [47], taking the alkane DPD particle as the reference. As a result, the mass of each benzene DPD particle was set to $m_{B}=1.7$, as was the force cut-off distance, $r_{c_{BB}}=1.17$. This defined each benzene particle as possessing number-average density of $\rho_{B}=0.65$. For benzene–benzene molecule interactions, a similar DPD force-field parameter of $a_{BB}=200$ was assumed. To validate the parameter selection, independent DPD simulations were performed and the normalized pair correlation function, g(r), was compared with experimental measurements. The pure liquid solvent simulation was performed in a cubic simulation box with the dimension of l≈8 in dimensionless DPD coordinates. Figure 1a depicts the correct behavior of g(r) for the simulated benzene liquid in comparison with experimental data [48,49].

The interactions between dissimilar particles in solutions require additional DPD force field parameters, namely, force cut-off distances, $r_{c_{aB}}$, and maximum repulsive force parameters, $a_{aB}$. The force cut-off between alkane and benzene particles were determined by an arithmetic mixing rule, $r_{c_{aB}}=\left(r_{c_{a}}+r_{c_{B}}\right)/2$. The DPD repulsive force parameter between dissimilar particles plays a crucial role in determining the thermodynamics and phase state of a multicomponent mixture. Groot and Warren [41] derived a connection between the repulsive force field parameter and the Flory–Huggins mixing parameter, χ, where $\chi \propto (a_{ii}-a_{ij})$. This equation was later developed into more sophisticated relations [50]; however, these models include simplifying assumptions that are not applicable to the present case. Note that the parameter χ was reported as −0.25 and 0.25 for polyethylene in hexadecane and polyethylene in benzene, respectively [51]. Assuming these values for χ, a direct comparison of DPD with molecular dynamics (MD) simulation results demonstrated that both solvents may be considered close to theta solvent conditions [43]. As a result, the trivial deviation in the repulsive force field parameter, $a_{aB}$, due to negligible differences in χ, is ignored. Therefore, a constant DPD repulsive force parameter was assumed as $a=a_{aa}=a_{BB}=a_{aB}$.

To verify the validity of the DPD predictions, the properties of paired DPD/MD polymer chains, N-334/$C_{1000}H_{2002}$, dissolved in hexadecane $N_{H}$-6 ($C_{16}H_{34}$) and benzene $N_{B}$-1$(C_{6}H_{6}$) were compared. The equilibrium simulations were performed in a small simulation box containing 8 and 10 N-334 chains dissolved in hexadecane and benzene solvents, respectively. Physical properties of the solutions in the quiescent state were calculated from statistically significant data, which were extracted from sufficiently long simulation times. These properties were compared with the results of equilibrium MD simulations from a recent publication by Nafar Sefiddashti et al., wherein the same polymer solutions were modeled using the Siepmann–Karaborni–Smit (SKS) united-atom model and integrated using the p-SLLOD equations of motion [43,52]. The SKS model, although originally developed to describe vapor–liquid equilibrium of linear alkanes, has proven to be widely applicable to PE melts and solutions in prior simulation studies.The details of the model validation will be discussed later in this section; however, the mapping strategy for obtaining DPD parameters directly from NEMD systems under both equilibrium and nonequilibrium conditions as developed by Nafar Sefiddashti et al. [42] was employed to produce the optimal DPD model solution.The DPD parameters used in this study are summarized in Table 1.

All DPD simulations were performed in the canonical (NVT) ensemble using the LAMMPS computing platform [53]. Newton’s equations of motions subjected to the DPD force field were integrated using the velocity-Verlet algorithm [41] with a suitably small time-step of $\Delta t=0.012\tau_{DPD}$ to prevent temperature deviations. The physical properties of the polymer solutions were directly extracted from simulation data. Characteristic properties such as the ensemble-averaged root-mean-squared end-to-end distance, ${\langle R^{2}\rangle }^{1/2}$, and the ensemble-averaged radius of gyration, ${\langle R_{g}^{2}\rangle }^{1/2}$, were obtained from the conformational analysis. Moreover, standard random walk relations were used to estimate the Kuhn segment length, $b=\langle R^{2}\rangle /L_{max}$, as roughly 16 Å or 4 DPD beads in length, and the number of Kuhn segments, $N_{K}=\langle R^{2}\rangle /b^{2}$, as approximately 235, where $L_{max}$ is the fully-extended contour length of the PE chains (about 3800 Å).

The dynamics of polymers in solutions change from Rouse dynamics to reptation as they overlap and entangle as the concentration ratio $c/c^{*}$ increases above unity, where *c* is polymer concentration and $c^{*}$ is the coil overlap concentration. Polymer concentration is calculated according to $c=(NN_{c})/V$, where *N*, $N_{c}$, and *V* are the number of beads per chain, number of chains, and volume of the simulation cell. Since the number density and mass of each polymer DPD particle were set to unity, the molar mass in DPD units is equivalent to the chain length. Moreover, the overlap concentration may be estimated by $c^{*}=N/(\frac{4}{3}\pi R_{g}^{3}$). The ratio $c/c^{*}$ was set at 20 for N-1000 in hexadecane and 15 for N-1000 in benzene, which implied that two significantly different entanglement numbers could be investigated.The volume fraction of polymer in each solution, ϕ, can also be calculated from $\phi =N_{p}/\rho_{a}V$, where $N_{p}$ (see Table 2) and $\rho_{a}$ (= 1 in DPD units) are the total number of polymer particles and number density, respectively, as 0.46 for n-hexadecane and 0.30 for benzene. The hexadecane solution is simulated at T≈391 K and the benzene solution at T≈353 K.

The longest time scale, associated with the disengagement time, $\tau_{d}$, of the entangled polymer solutions, was estimated by fitting sums of two and three-term exponential functions to the ensemble-averaged autocorrelation function as $\langle u\left(\tau \right)\cdot u\left(t+\tau \right)\rangle =\sum c_{i}\exp\left(-2\pi t/\tau_{i}\right)$ [54], where u=R/|R| is the unit end-to-end vector and the $c_{i}$’s are fitting parameters. The results from NEMD simulations of polyethylene liquids [55,56,57], along with theoretical relationships for reptation-based calculation of the disengagement time, $\langle u\left(0\right)\cdot u\left(t\right)\rangle =\sum_{p:odd}\frac{8}{p^{2}\pi^{2}}\exp\left(-p^{2}t/\tau_{d}\right)$ [4,58], served to validate the method. Moreover, the longest characteristic time of the chains under flow, $\tau_{d_{f}}$, and tumbling/rotation time, $\tau_{r_{f}}$, were calculated via the equation $\langle u\left(\tau \right)\cdot u\left(t+\tau \right)\rangle =A\exp\left(-2\pi t/\tau_{d_{f}}\right)\cos\left(-2\pi t/\tau_{r_{f}}\right)$, where *A* is a fitting parameter [59]. (Note that these two time scales are Wi-dependent, decreasing in magnitude with increasing shear rate.) Two additional characteristics time scales are associated with tube segment stretching and entanglement dynamics, namely, the Rouse time, $\tau_{R}\equiv \tau_{d}/3Z$, and the entanglement time, $\tau_{e}\propto \tau_{R}/Z^{2}$, where *Z* is defined as the ensemble average number of entanglements per chain at equilibrium. Note that $Z={\langle L\rangle }^{2}/a_{t}={\left(N_{K}b\right)}^{2}/a_{t}^{2}={\langle L\rangle }^{2}/\langle R^{2}\rangle$, where $a_{t}$ and 〈L〉 are the equilibrium tube diameter and the average primitive chain contour length, respectively. The Z1-code of Kröger [60,61,62,63] was used to perform the network topological analysis and calculate relevant length scales, such as *L* and $a_{t}$, at equilibrium. The number of kinks per chain, $Z_{k}$, which is reported by Z1-code and has been shown to be proportional to Z by the ratio $Z_{k}/Z\approx 2$ at equilibrium [55,56,57,61,64,65] is an alternative measure for network entanglement. However, the validity of this ratio along with physical definitions of *Z* and $a_{t}$ under nonequilibrium flow conditions remains uncertain. Thus, we henceforth report the number of kinks per chain, $Z_{k}$, since this quantity has an unambiguous geometric definition in the Z1 code.

The stress tensor, T, was calculated over all polymer particles within the simulation cell, and on some occasions, over polymer particles in selected layers of the simulation cell to generate a stress profile within the flowing liquid. The total stress was calculated according to the Irving–Kirkwood equation [66],

(7) $$T=\frac{N_{p}k_{B}T}{V}\delta +\frac{\langle \sum_{i=1}^{N_{p}}r_{i}F_{i}^{tot}\rangle }{3V}\;,$$

where the first term quantifies the kinetic effects on the hydrodynamic pressure and the second term represents the virial coefficient associated with the DPD force-field model.

**Figure 2:**
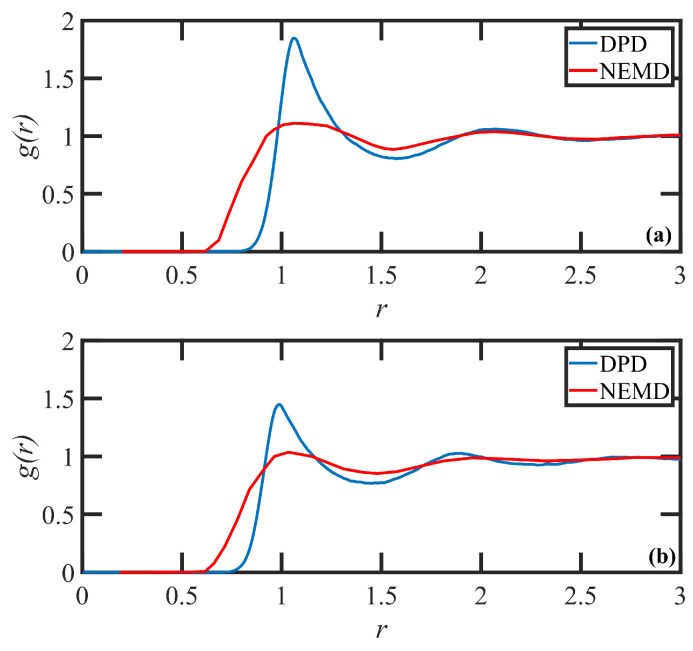
The polymer-solvent pair correlation functions, g(r), for (**a**) N334-benzene solution and (**b**) N334-hexadecane solution compared to results of a $C_{1000}H_{2002}$ MD simulation. Note that the MD data were normalized to DPD units for comparison.

As mentioned above, the properties of simulated paired DPD/MD (N-334/$C_{1000}H_{2002}$) polymer solutions at equilibrium were compared to examine the validity of the DPD model. The first solution was PE N-334 in hexadecane at 14.5 $c/c^{*}$, and the second one was the same polymer at 13.5 $c/c^{*}$ in benzene. A direct comparison of the pair correlation function, g(r), for both polymers at equilibrium illustrates a good overall agreement between paired DPD/NEMD systems, as displayed in Figure 2. The peak maxima are accurately described, although the DPD peaks are somewhat narrower due to the coarse-grained lumping of multiple methylene groups into DPD particles. Although the agreement between the models demonstrates the microscale compatibility of the two solution systems, this measure alone may not guarantee the satisfactory accuracy of polymer dynamics under nonequilibrium conditions. Therefore, to ensure the correct dynamic behavior of polymers, steady-state and large time-scale properties of the system, such as the disengagement time and the center-of-mass diffusivity between paired DPD/MD solutions may also be examined—see Figure 1b. To this end, the center-of-mass mean-squared displacement versus time (which are scaled by squared ensemble-averaged radius of gyration and disentanglement time, respectively) were simulated for the N-334 PE DPD system in both solvents and compared to similar quantities calculated from equilibrium molecular dynamics simulations of PE solutions of $C_{1000}H_{2002}$. Figure 1b shows the satisfactory agreement for both of the solutions. Note that we used scaled mean-squared displacement as a measure to fine-tune the interaction cut-off distance between dissimilar particles. Moreover, the topological analysis demonstrates an agreement between the number of kink entanglements per chain for the pair DPD/MD test chains. Specifically, the pair N-334/$C_{1000}H_{2002}$ in hexadecane solution has $Z_{k}=15$ and the pair N-334/$C_{1000}H_{2002}$ in benzene solution has $Z_{k}=10$. Overall, the results confirm that the proposed DPD model is capable of capturing the solution dynamics reliably. For further details regarding the MD results, see Ref. [43].

## 3. Results and Discussion

### 3.1. Quiescent Properties

Equilibrium and nonequilibrium DPD simulations were performed of two entangled polymer solutions containing linear macromolecular chains of 1000 ($C_{3000}H_{6002}$) DPD particles dissolved in hexadecane and benzene at concentrations of 20 $c^{*}$ and 15 $c^{*}$, respectively. Table 2 displays the sizes of the simulation cells at equilibrium for the two PE solutions. The cell sizes were chosen to remain large enough to avoid any self-interaction of individual chains with their periodic images under nonequilibrium conditions. The solutions contained 240 and 370 PE chains dissolved in 350,000 benzene and 70,230 hexadecane molecules, respectively. The physical properties of the N-1000 polymer chains in the quiescent equilibrium state were obtained from the DPD simulations, as summarized in Table 3. Most of the topological properties of the N-1000 chains are similar in both solutions, except the average number of entanglements per chain is approximately 50% higher in the oligomeric solvent. Hence, solvent quality is similar in both solutions, and from the relevance of random-walk statistics, both solutions are deemed to be at near-theta condition.

### 3.2. Steady-State Shear Flow: Rheology and Network Topology

Step-strain startup of shear flow simulations were conducted by imposing a specific value of shear rate to an equilibrated solution system under quiescent conditions and then tracking the evolution of the system phase-space trajectory until a steady-state flow condition had been attained. Statistically meaningful values were obtained for each of the rheological and topological parameters under steady-state conditions. In the simulations, *x* is the flow direction, *y* is the gradient direction, and *z* is the neutral direction with respect to the coordinate system of the simulation box. The two solution systems were exposed to a wide range of shear rates, represented in terms of the Weissenberg number, $Wi=\tau_{d}\dot{\gamma }$, where $\dot{\gamma }$ is the shear rate (also in DPD units), spanning 0.5≤Wi≤ 12,000 for the benzene solution and 3≤Wi≤ 10,000 for the hexadecane solution. The initial (t=0) configurations used for startup constituted quiescent equilibrated systems for each solution with a long-time homogeneous polymer concentration distribution throughout the simulation cell. However, it should be noted that thermal fluctuations cause a continuous slight change in the polymer concentration distribution inside the simulation box; it will be demonstrated later that these mild fluctuations do not induce inhomogeneities in the entanglement network or bulk rheological and topological properties.

The steady-state properties of the system under shear flow were determined over the ranges of Wi stated above. The steady shear properties of the concentrated entangled solutions exhibited four distinct characteristic dynamic regions based on their characteristic time scales and flow microstructure, as observed in prior NEMD simulations of linear, monodisperse polyethylene melts [11,55,56,57,67], i.e., ranges of shear rate roughly delineated by $\dot{\gamma }<\tau_{d}^{-1}$, $\tau_{d}^{-1}<\dot{\gamma }<\tau_{R}^{-1}$, $\tau_{R}^{-1}<\dot{\gamma }<\tau_{e}^{-1}$, and $\dot{\gamma }>\tau_{e}^{-1}$. (Note, however, that no steady-state or transient shear banding occurred at $\dot{\gamma }>\tau_{e}^{-1}$, and hence the fourth region will not be discussed in this article.) A number of physical properties were calculated from the simulation output to characterize the rheological and topological behavior of the polymer solutions, including the polymer contribution to the shear stress, $\sigma_{xy}^{p}$, the viscosity, $\eta_{p}=\sigma_{xy}^{p}/\dot{\gamma }$, the average number of entanglement kinks per chain, $Z_{k}$, and the tube segmental orientation tensor, S=〈uu〉. Note that tube segments were defined by dividing the N-1000 chain into 20 segments (50 beads per segment) for both solutions. Also note that only the polymer contribution to the shear stress (and normal stresses) is reported below; total stress, including all polymer and solvent interactions, displayed qualitatively similar behavior to that of the polymer stress contribution and was quantitatively shifted upward by an amount dictated by an approximately Newtonian viscosity. (Note that the effective Wi applied in the simulations are large with respect to the reciprocal time scales of the polymer molecules but not with respect to those of the solvents.) Hence, only the polymer contribution to shear stress is reported below since it is most directly related to the macromolecular configurational dynamics that induce shear banding.

**Figure 3:**
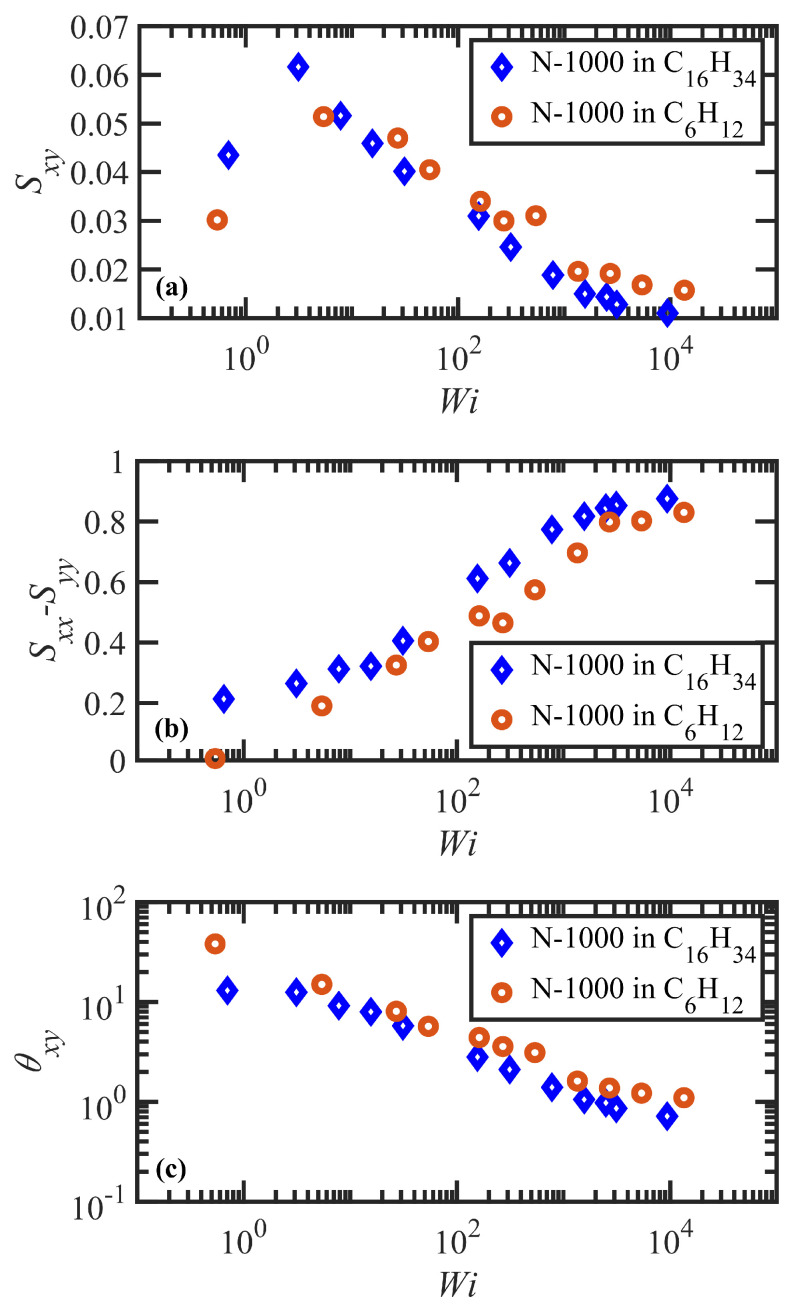
Components of the tube orientation tensor, S=〈uu〉, where u is the unit vector based on the segmental end-to-end vector, plotted versus Wi: (**a**) $S_{xy}$ and (**b**) $S_{xx}-S_{yy}$. Panel (**c**) displays the behavior of $\theta_{xy}$, the average angle of tube segment orientation, defined as the angle (measured in degrees) between the eigenvector associated with the largest eigenvalue of S and the flow direction in the flow-gradient (*x*-*y*) plane.

**Figure 4:**
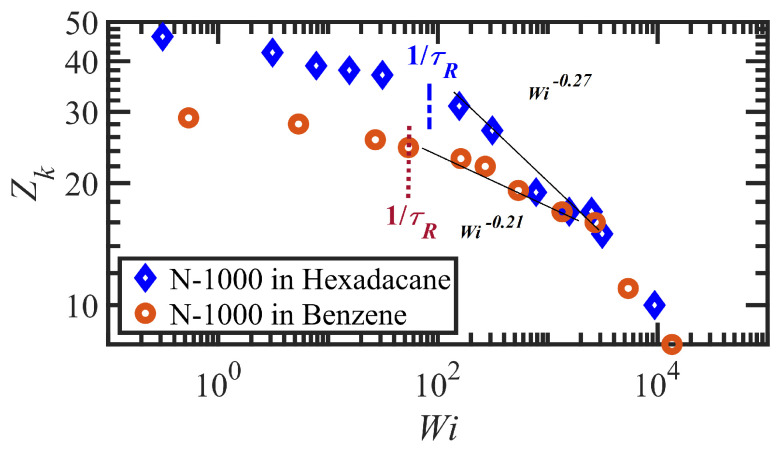
Average number of kink entanglements per chain, $Z_{k}$, vs. Wi for both solutions undergoing steady-state shear flow. Shear rates corresponding to $1/\tau_{d}$ are represented at $Wi=10^{0}$ and those corresponding to $1/\tau_{R}$ are indicated by the dotted vertical lines. The lowest Wi data point of each solution indicates that $Z_{k}$ retains its equilibrium value at Wi<1.

**Figure 5:**
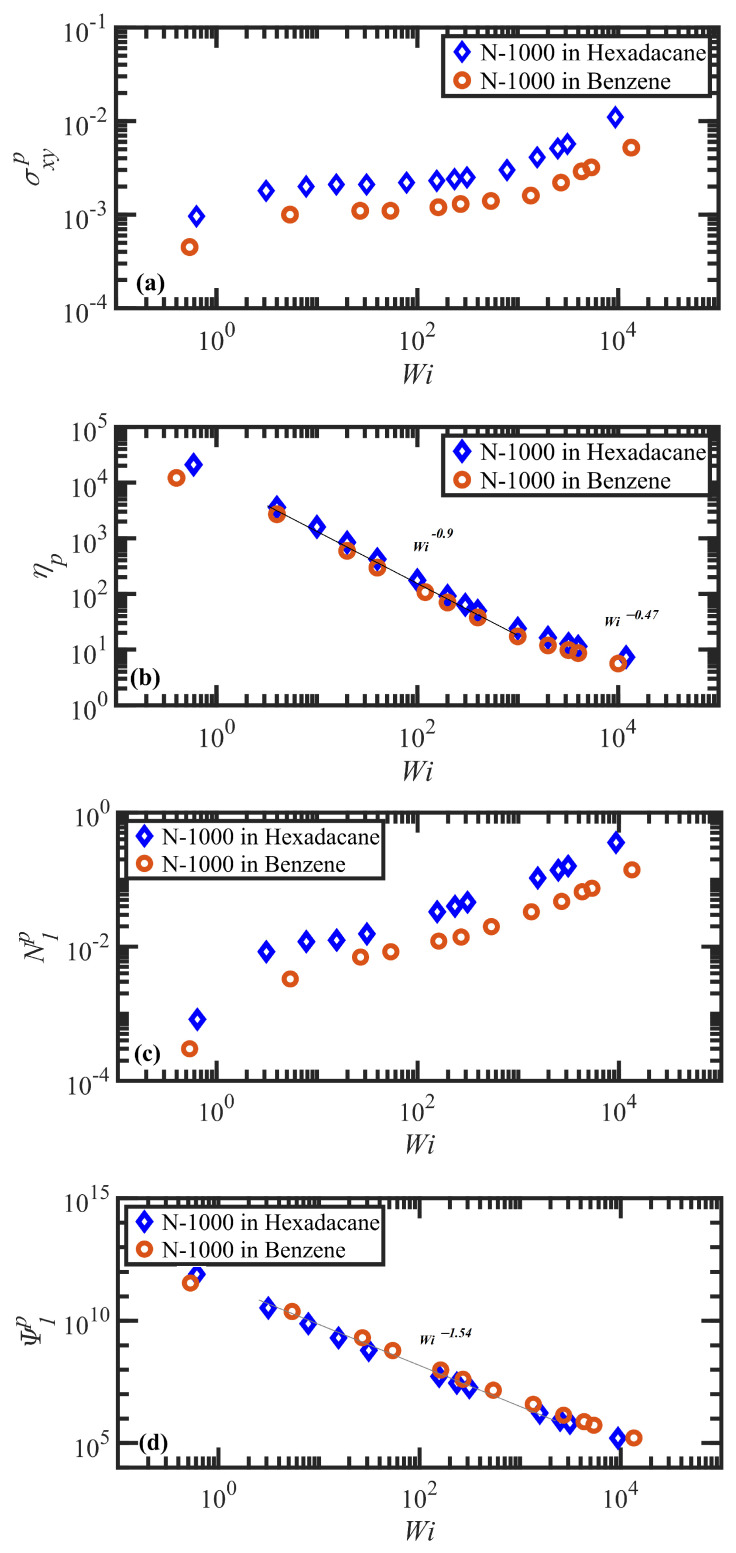
Steady-state bulk rheological behavior of the two PE solutions, N-1000 in hexadecane and N-1000 in benzene: (**a**) steady-state shear stress, $\sigma_{xy}^{p}$, plotted versus Wi; (**b**) steady-state viscosity, $\eta_{p}$, versus Wi; (**c**) steady-state first normal stress difference, $N_{1}^{p}$; (**d**) steady-state first normal stress coefficient, $\Psi_{1}^{p}\equiv \left(\sigma_{xx}^{p}-\sigma_{yy}^{p}\right)/{\dot{\gamma }}^{2}$. All quantities are displayed in DPD units. Error bars on stress values were smaller than the size of the symbols in all cases and were therefore omitted.

**Figure 6:**
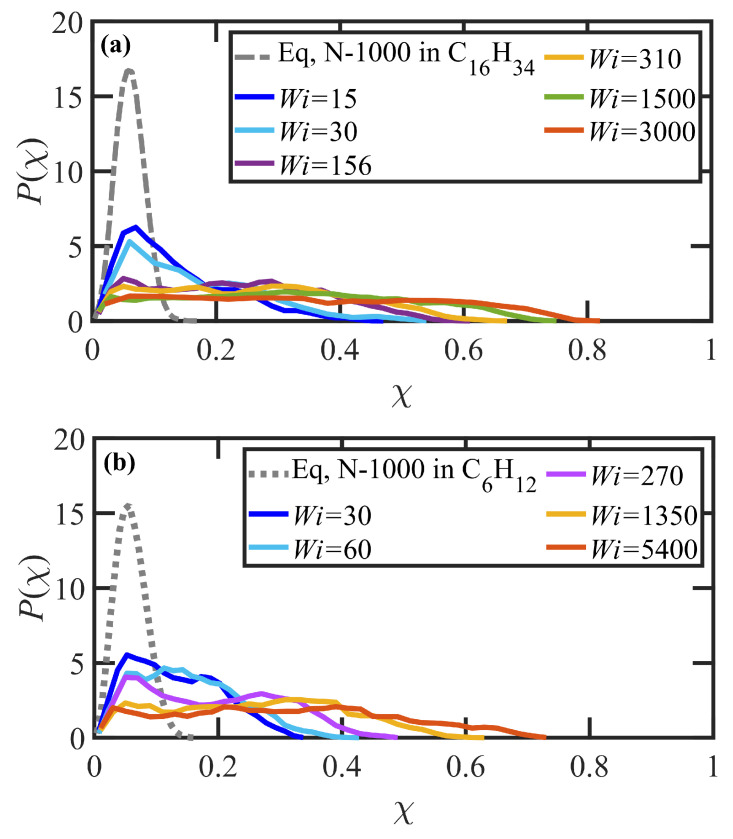
Probability distribution function of chain fractional extension, χ, for (**a**) the N-1000 in hexadecane solution, and (**b**) N-1000 in benzene solution, subjected to steady-state shear flow at selected Wi within different flow regimes. Eq denotes the quiescent equilibrium state.

The first region is the linear viscoelastic flow regime encompassing $\dot{\gamma }<\tau_{d}^{-1}$ or Wi<1. Over the slow deformation of the fluid, the polymer molecules can essentially retain their equilibrium configurations since the time scale of the deformation is longer than that of the tube disengagement time. In other words, the chains have adequate time to diffuse through the tube network to maintain their most favorable entropic configurations, although overall the tube network reorients (but does not stretch) along a preferred direction with respect to the flow direction. This reorientation manifests as a steep increase in the $S_{xy}$ component of the tube orientation tensor, as shown in Figure 3a, and a decrease in the average tube orientation angle, $\theta_{xy}$, as displayed in Figure 3c. (Note that obtaining steady-state data within this low Wi region is very computationally expensive, and thus only a single data point for each solution at Wi<1 is presented in the following figures. Since nothing unusual or interesting was observed in this Wi region beyond the expected linear viscoelastic behavior described in previous simulations of similar PEs [55,56,57], the authors focused on obtaining a sufficient number of data points in the nonlinear viscoelastic regime where the simulations attain steady state much faster. Therefore, some of the trends discussed in this paragraph are merely inferred from the previous studies and typical behavior of linear viscoelastic fluids.) Since there is no tube deformation in this regime, however, the diagonal components of S are not affected by the flow hydrodynamics, as depicted in Figure 3b. Furthermore, the average number of entanglement kinks, $Z_{k}$, also remains near its equilibrium value—see Figure 4. The shear stress $\sigma_{xy}^{p}$ increases linearly in this Wi range, providing a constant value of viscosity $\eta_{p}$ of about $10^{4}$ (in DPD units) for both solutions—see Figure 5a,b. The small increase in $S_{xx}-S_{yy}$ depicted in Figure 3b with Wi is proportional to a similar slight increase in the first normal stress difference, $N_{1}^{p}=\sigma_{xx}^{p}-\sigma_{yy}^{p}$, as shown in Figure 5c, whereas the first normal stress coefficient ($\Psi_{1}^{p}\equiv \left(\sigma_{xx}^{p}-\sigma_{yy}^{p}\right)/{\dot{\gamma }}^{2}$) is approximately constant at very low shear rates—see Figure 5d. In this flow regime, the probability distribution functions (PDFs) of fractional chain extension, defined for each macromolecule as $\chi =\left|R|/\right|R_{max}|$, deviate little from their equilibrium distributions, which are displayed in Figure 6. Reptation theory adequately describes the dynamics of the polymer chain properties and rheological behavior in this linear viscoelastic regime, as noted previously for entangled polyethylene melts subjected to steady-state shear flow [55,56,57,68].

The second flow regime occurs within the shear rate range of $\tau_{d}^{-1}<\dot{\gamma }<\tau_{R}^{-1}$, corresponding to 1<Wi<75 and 1<Wi<51 for the N-1000 in hexadecane and benzene solutions, respectively. The $S_{xy}$ component of the tube orientation tensor displays a nonmonotonic behavior, increasing substantially up to a maximum around Wi≈3–5 and subsequently decreasing with $\dot{\gamma }$. This nonmonotonic behavior is caused by the competing effects of increased degree of alignment with increasing Wi, which increases the average $\langle u_{x}u_{y}\rangle$ as more segments become aligned at the primary eigenvalue, whereas the decrease in the angle of alignment $\theta_{xy}$ with $\dot{\gamma }$ reduces $u_{y}$ to zero at high Wi; i.e., individual dyadic elements $u_{x}u_{y}$ vary with $\theta_{xy}$ as $\cos\theta_{xy\;}\sin\theta_{xy}$, implying a decrease from 0.5 to 0 as $\theta_{xy}$ transitions from 45° to 0° with increasing Wi—see p. 8133 of Ref. [69] for a more detailed explanation. Complementing the increase in tube segment orientation (which remains the dominant dynamical mechanism within this range of $\dot{\gamma }$), the tube segments also begin to stretch as indicated by the increase of $S_{xx}-S_{yy}$ in Figure 3b. (See also plots of the tube stretch variable, λ, for similar polyethylene melts in Figure 61d of Ref. [67].) The kink entanglement number begins to decline in this Wi range, indicating an initial degradation of the entanglement network, as also observed for the entangled polyethylene melt simulations [55,56,57,70,71]. The destruction of the entanglement network results in an increase in chain mobility as the average tube diameter increases, leading to the onset of individual chain tumbling dynamics at Wi in the range of 5–10. Within this region, $Z_{k}$ scales according to a power-law behavior with Wi of exponents −0.078 and −0.061 for the N-1000 hexadecane and benzene solutions, respectively. Throughout the range Wi>5 after the maximum in the $S_{xy}$ profile, the shear stress establishes a constant plateau value for both solutions, which is a result of the steady decrease in entanglement network constraints and the onset of chain tumbling. As a result, the viscosity exhibits an initial shear-thinning behavior that scales as $\eta_{p}\propto Wi^{\alpha }$ with α=−0.9 for both solutions. The first normal stress difference increases monotonically in this Wi region, whereas $\Psi_{1}^{p}$ decreases with Wi according to a power-law expression with exponent of −1.54 for both solutions. The corresponding PDFs of fractional chain extension of Figure 6 broaden substantially toward more extended chains within this Wi regime, which is indicative of the stretching of the tube segments and also the onset of chain tumbling that results in quasiperiodic extension/retraction cycles of the individual macromolecules [54,59,67,72].

**Figure 7:**
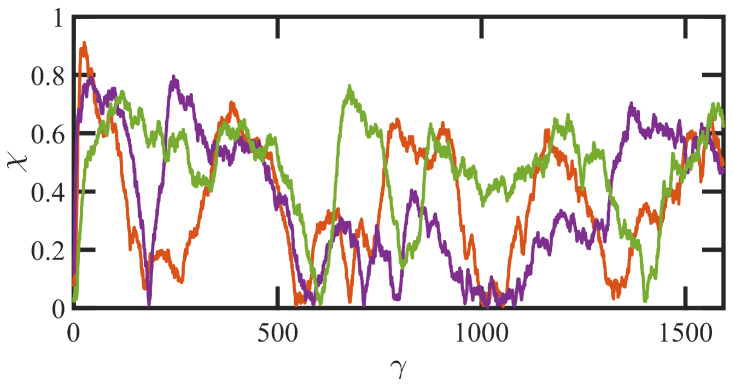
The temporal evolution of fractional extension vs. strain for several individual molecules of the PE solution N-1000 in hexadecane undergoing startup of shear flow at Wi=1500. The quasiperiodic cycles of chain extension and retraction reveal the dominant tumbling dynamics. Note that each color line represents a randomly selected chain.

At higher flow rates, i.e., $\tau_{R}^{-1}<\dot{\gamma }$ corresponding to Wi>75 (hexadecane) and 51 (benzene), the flow enters the third shear-rate regime in which tube stretching dictates the flow dynamics, replacing segmental orientation as the dominant relaxation mechanism. As the individual molecules begin to extend significantly with the relaxation of the entanglement network constraints, they experience quasiperiodic extension/retraction cycles at frequencies that scale with shear rate and which can require 100’s of strain units to complete a full cycle—see Figure 7. $S_{xy}$ continues its decline with Wi, finally plateauing out at a low value that is consistent with the approach of $\theta_{xy}$ to value slightly larger than 0°, indicating average segmental alignment mostly along the flow direction at high Wi. Figure 3b displays the steep increase in $S_{xx}-S_{yy}$ as a quantitative measure of segmental stretching, which induces a commensurate relaxation of the entanglement network as evident in Figure 4. The decrease in the number of entanglement kinks scales with a power-law exponent within the range of −0.21 to −0.27 at high Wi. As the shear rate increases beyond $\tau_{R}^{-1}$ in Figure 5a, the shear stress begins to increase above the plateau level, with the viscosity data exhibiting a smaller slope of power-law exponent −0.47 at very high Wi. $N_{1}^{p}$ experiences a steady increase with Wi, indicative of the increased extension of the macromolecules, and $\Psi_{1}^{p}$ continues to decrease steadily with increasing shear rate with a similar scaling exponent (−1.54) as in the second shear-rate region. It is evident that the reduction in the number of molecular entanglements results in more extended molecules and more frequent chain tumbling dynamics, which are manifested in the broad PDFs of relative extension χ of Figure 6, ranging evenly from very small values associated with retracted chains to high values of relatively extended molecules.

As described above, the steady-state behavior of the simulated linear monodisperse PE solutions exhibits a monotonic flow curve, $\sigma_{xy}^{p}$ vs. Wi, as depicted in Figure 5a. This behavior is in qualitative agreement with prior experimental and simulation data of entangled polymer solutions, exhibiting essentially a monotonic stress curve with a very small positive slope in the shear-rate region where the stress plateau is established, wherein the dominant flow mechanism shifts from segmental orientation to deformation and disintegration of the tube network. A detailed discussion of transient shear-band formation and reverse flow in startup of shear flow of polymer solutions is presented in the next section, in which it is demonstrated that shear banding occurs shortly after startup of shear flow due to microstructural inhomogeneity in the Wi region of the stress plateau. This inhomogeneity of the inherent polymer microstructure results in a commensurate inhomogeneity in the polymer concentration profile, which persists throughout the duration of the shear-band lifetime.

### 3.3. Startup of Shear Flow: Transient Shear Banding, Reverse Flow, and Concentration Fluctuations

**Figure 8:**
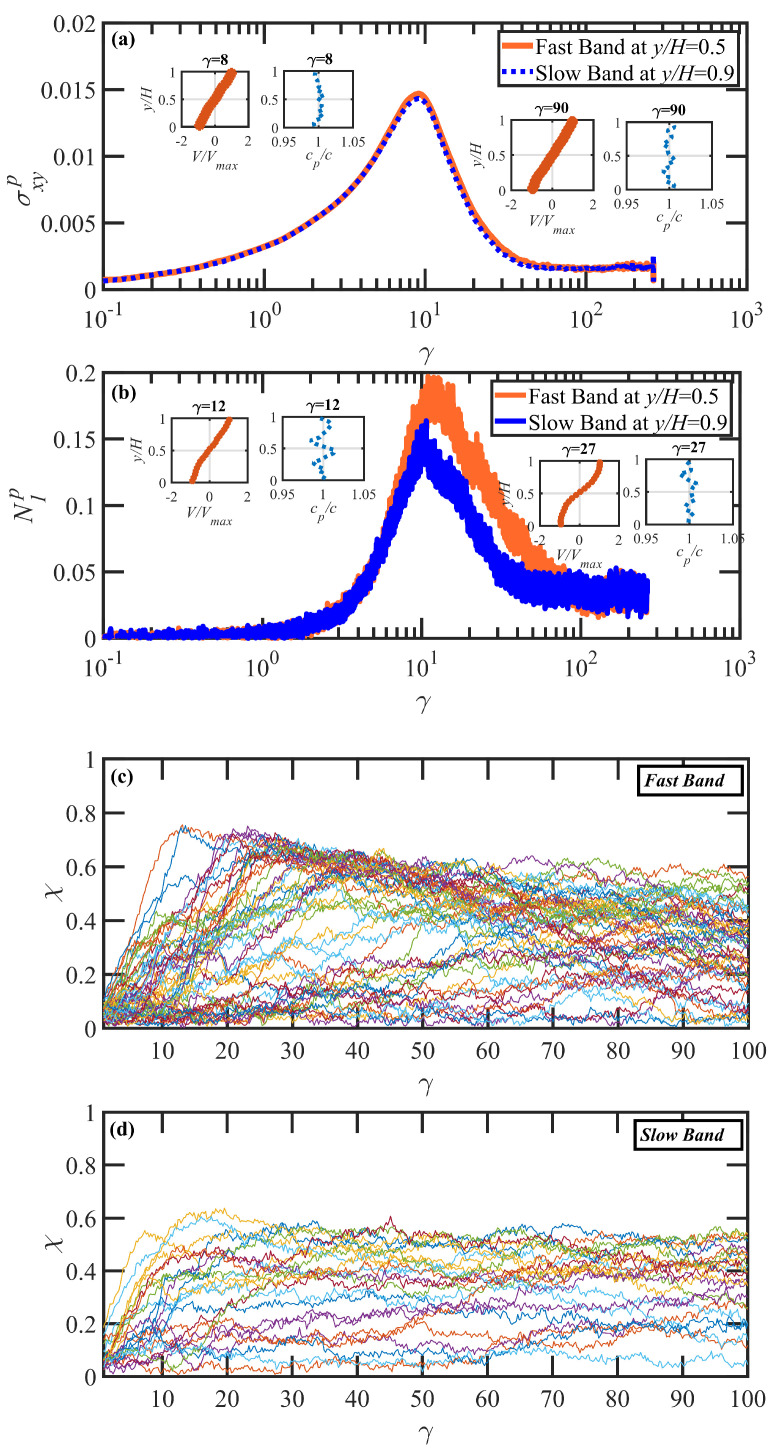
The temporal evolution (in strain units) of (**a**) shear stress, $\sigma_{xy}^{p}$, and (**b**) first normal stress difference, $N_{1}^{p}$, vs. strain units within the fast (layer centered at y/H=0.5) and slow (layer centered at y/H=0.9) shear bands for PE solution N-1000 in benzene undergoing startup shear flow at Wi=1350—see also Figure 13c. The insets in panels (**a**,**b**) display the scaled instantaneous velocity profile, $V/V_{\max}$, and scaled instantaneous polymer concentration, $c_{p}/c$, spanning the scaled simulation cell dimension (y⁄H) in the *y*-direction at specific values of applied strain. Note that layer velocities are reported relative to the center of the simulation cell, such that the velocity is evaluated as negative in the bottom half of the cell. Panels (**c**,**d**) present the temporal evolution of fractional extension (χ) of individual molecules (each assigned a random color) in the fast band (**c**) and slow band (**d**).

Upon application of a constant shear rate to the quiescent solution at time t=0 via an appropriate steady motion at the simulation cell boundaries (y=0,H), a transverse shear wave propagates in the gradient direction through the simulation cell on a time scale of $t_{p}=H^{2}\rho /\eta$, where *H* is the dimension of the simulation box in the *y*-direction [67]. Consequently, in the present simulations the shear profile is experienced throughout the cell within the span of a few time increments at all Wi. This implies that the shear stress is homogeneous within the simulation box at all greater times, which can be readily verified by subdividing the cell into a given number of layers arranged perpendicularly to the gradient direction. (This will be demonstrated below—see Figure 8a. The interested reader can also refer to Figure 5 of Ref. [46].)

The steady-state shear stress is a monotonically increasing function of Wi, as observed in Figure 5a above, although the slope in the plateau region at intermediate Wi is only slightly positive. This implies that at each simulated value of shear rate, there is a unique value of shear stress associated with the applied Wi. Accordingly, the system will settle into a unique, homogeneous microstructural state as the liquid approaches its steady-state condition for each respective value of applied shear rate. At low and high shear rates, where $\sigma_{xy}^{p}$ varies significantly with Wi, the startup behavior is rather uninteresting as the established shear stress effectively imposes a unique and homogeneous shear rate throughout the simulation cell. However, for startup of shear flow for Wi in the plateau region, ranging roughly from Wi∈[1,1000], the variation in shear stress with Wi is only nominal; hence, widely differing applied shear rates induce only mild changes in shear stress. Therefore, under startup conditions of shear flow at shear rates within the plateau region of the $\sigma_{xy}^{p}$ vs. Wi profile, the stochastic nature of the simulation (see Equations (1) and (4)) can produce multiple microstructural states that possess approximately the same value of shear stress but widely differing Wi values, inhomogeneously arranged along the gradient direction. These microstructural states are generated by the individual molecular dynamics of the chains in localized neighborhoods of the sample, each possibly corresponding to a vastly different Wi than those at other locations within the cell. During this phase of startup, the liquid can experience transient shear banding as the system zeroes in (or converges to) a steady-state homogeneous liquid experiencing a uniform velocity profile as defined by the imposed motion of the boundaries. As it turns out, these temporary and vastly different microstructural states often result in other curious dynamical artefacts, such as reverse flow and flow-induced concentration fluctuations, which disappear once the system finally attains steady state. This entire process from flow onset to steady state can span several hundreds of strain units.

Shear banding observed in viscoelastic fluids can be induced by elastic and viscous instabilities associated with the microstructural configuration and entanglement network caused by inhomogeneities associated with the stochastic nature of individual molecular dynamics under flow [11,12,73,74,75]. The nature of the inhomogeneity is further complicated by particle–particle interactions and concentration dependencies in two-fluid systems, particularly solutions. Furthermore, the monotonicity of the shear stress profile in solutions raises a fundamental question about the origin of this phenomenon, which cannot be explained by classical constitutive instability. As a result of prior analyses [11,12], shear banding is thought to be primarily a stochastic process resulting from the unpredictable dynamics of individual chains in localized flow regions [11]. It is important to note that, despite this picture being rather general, numerous environmental factors (such as temperature and instrument compliance) may contribute to this phenomenon.

In recent experimental and theoretical studies, it has been suggested that shear banding in solutions is induced by fluctuations in polymer concentration [35,36,76]. It is therefore critical to investigate the complex interaction between flow-driven entanglement network inhomogeneity and concentration fluctuations in order to understand the dynamic origins of shear-banded structures in greater detail. Thus, the temporal evolution of the velocity and concentration profiles throughout the startup flow process (in the gradient direction) have been extracted from the simulation output and examined through microstructural analysis. The velocity and concentration profiles were extracted by averaging the instantaneous velocities in the flow direction over time intervals of $0.1\tau_{d_{f}}$ for particles within each of 20 layers along the gradient (*y*-direction). These data demonstrate that both N-1000 hexadecane and benzene solutions developed transient shear-banded structures at applied shear rates within the range of $\tau_{d}^{-1}<\dot{\gamma }<\tau_{e}^{-1}$.

Figure 8a,b depict the temporal evolution of shear stress, $\sigma_{xy}^{p}$, and first normal stress difference, $N_{1}^{p}$, in strain units for the fast and slow bands of the PE N-1000 in benzene solution undergoing startup shear flow at Wi=1350. These quantities were evaluated within layers of thickness 0.2y/H perpendicular to the gradient direction centered at y/H=0.5 and y/H=0.9 within the fast and slow bands, respectively. These particular layers were chosen because they are fully contained within distinct shear bands that develop within the system at around 12 strain units and ultimately dissipate after 90 strain units. These shear bands are recognized from the velocity profiles within this range of strain, as shown in the insets of Figure 8a,b—in particular, see the inset in Panel (b) at γ=27. The startup shear stress profile increases at small strain, and the velocity profile in the gradient direction is uniform, with a slope corresponding to the imposed value of shear rate. $\sigma_{xy}^{p}$ exhibits an overshoot with a maximum at γ≈8, at which time the velocity profile in the gradient direction remains uniform and then subsequently decreases with strain, gradually settling down to its steady-state value. Note that the shear stress is uniform throughout the simulation cell, as demonstrated in Figure 8a for the fast and slow bands where the shear stress is essentially the same at all times within both layers.

The first normal stress difference also increases with strain during the initial stage of startup, where the velocity profile is uniform. In this range of strain units, $N_{1}^{p}$ is constant across the cell in the gradient direction. $N_{1}^{p}$ then experiences an overshoot with a maximum at γ≈12, which is the point of incipient shear-band generation. Note that the maximum of $N_{1}^{p}$ typically occurs about 3–5 strain units after the maximum in $\sigma_{xy}^{p}$. After passing through the maximum, the first normal stress difference becomes inhomogeneous, with vastly different values within the distinct shear bands, with the fast band possessing the higher value, indicating a greater degree of molecular extension relative to molecules located within the slow band. $N_{1}^{p}$ decreases with γ, and eventually settles into its steady-state value at γ≈100. Note that this is significantly greater that the strain required to attain steady state (γ≈50) of the shear stress. By this time, the shear bands have completely dissipated, and $N_{1}^{p}$ again becomes uniform in the gradient direction. All of these observations are consistent with similar behavior of $N_{1}^{p}$ for an N-1000 PE melt as described by Boudaghi et al. [11].

The insets of Figure 8a,b also display the scaled concentration profile ($c_{p}/c$, where $c_{p}$ is the layer polymer concentration and *c* is the bulk polymer concentration) across the simulation box in the gradient direction at selected values of applied strain. At very low strains and very high strains, the concentration profile in the *y*-direction is essentially constant. However, at γ=12, the formation of the shear band is apparently accompanied by a significant concentration fluctuation (of up to ±3% magnitude) as the microstructural instability propagates through the liquid. These concentration fluctuations persist for the duration of the transient shear-banding period, but they eventually dissipate as the shear bands give way to a uniform velocity profile—see Figure 13c for additional concentration profiles for this system at other strain values. These concentration fluctuations appear to be the result of the individual chain dynamics within various regions of the flow cell, which can experience varying configurational evolution upon startup of flow due to the stochastic nature of the process. Hence, during the period of shear-band formation, the configurational rearrangements of the localized groups of individual molecules induce a migration of polymer particles that results in distinct layers of relative accumulation and depletion. Although impossible to prove conclusively from the present simulations, it appears evident that the inhomogeneous concentration profile is a byproduct of the localized microstructural evolution under startup of shear flow within the liquid, rather than an integral component of the system dynamics and instability that lead to transient shear banding.

The individual molecular dynamics of all molecules with centers of mass that are located within the fast and slow layers (as defined above) at the moment of shear-band formation (γ=12) are displayed as fractional extension (χ) vs. γ in Figure 8c,d. In these panels, each individual chain was assigned a random color, and its fractional extension was plotted from the initiation of startup (γ=0) to steady-state behavior at γ=100. Under steady-state conditions, both bands are inhabited by molecules with fractional extension varying widely from 0.05 to 0.6, as expected from the broad PDF of Figure 6b (yellow curve). Upon startup of shear flow, the low-strain monotonically increasing behavior of the molecules in both bands is essentially the same up to γ≈12, where the maximum in the $N_{1}^{p}$ overshoot occurs. Subsequently, chains within the slow band assume roughly constant values of χ, indicating a flow-aligning behavior, whereas those within the fast band continue to extend to higher values of χ out to γ≈25, at which time the chains begin to experience extension/retraction quasiperiodic tumbling cycles. (Keep in mind that a complete cycle can take several hundreds of strain units—see Figure 7; therefore, only the first phase of a full tumbling cycle is evident in Figure 8c.) The individual tumbling cycles are mostly out of phase with each other, thus evidencing a rather muddled plot of χ vs. γ at later times. As the shear bands disintegrate and merge into a uniform velocity profile at γ>100, the individual chains all assume a similar behavior, which gradually resembles that of the final strain units depicted in Panel (c); i.e., the molecules in the slow band also exhibit quasiperiodic tumbling cycles at γ>100 (not shown).

**Figure 9:**
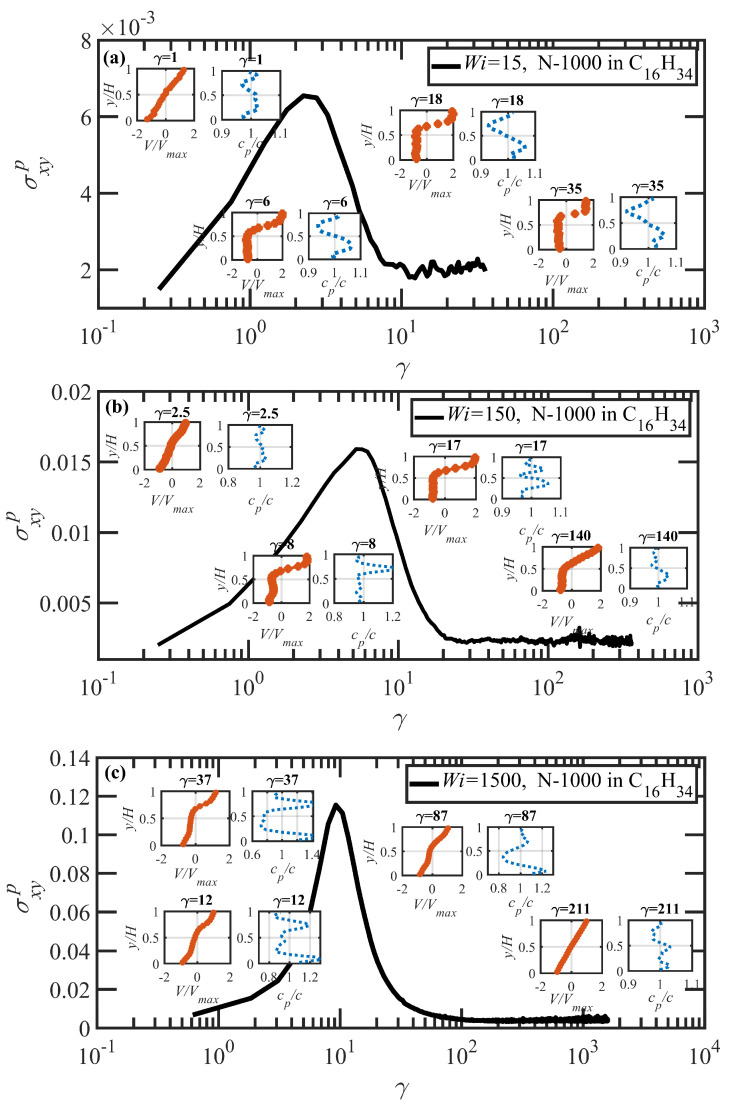
The temporal evolution of shear stress vs. strain units for PE solution N-1000 in hexadecane undergoing startup shear flow at (**a**) Wi=15, (**b**) Wi=150, and (**c**) Wi=1500. The insets display the scaled instantaneous velocity profiles, $V/V_{\max}$, and scaled instantaneous polymer concentration profiles, $c_{p}/c$, spanning the scaled simulation cell dimension in the *y*-direction (y⁄H) at specific values of applied strain.

Figure 9 displays simulation data from startup of shear flow simulations of the N-1000 PE in hexadecane solution at several values of Wi (=15, 150, and 1500) spanning the shear-rate range associated with the shear stress plateau evident in Figure 5a. The main graph of each panel depicts the shear stress versus strain behavior after startup of flow, with a substantial overshoot being observed in each case. The strain value associated with the maximum of the shear stress overshoot, $\gamma_{max}$, increases with Wi, assuming values of approximately 2.5 at Wi=15, 5 at Wi=150, and 9 at Wi=1500. Further analysis reveals that $\gamma_{max}$ scales with shear rate as ∼$Wi^{\;0.338}$. This scaling agrees with the power-law exponent of 1/3 obtained by prior theoretical studies [77] and atomistic simulations (see Figure 10b of Ref. [69]). The strain value associated with the maximum of the $N_{1}^{p}$ overshoot (not shown) was determined to be approximately 5.5 at Wi=15, 8 at Wi=150, and 12 at Wi=1500.

The insets in Figure 9 show the temporal evolution of the normalized velocity profile (red data points) and scaled local polymer particle concentration (dotted blue lines) spanning the *y*-direction at different time instants (strain values) after startup of shear flow. The pattern for the evolution of the velocity profile is very similar to that of similar simulations composed of entangled PE melts [11,12,13,15]. Specifically, starting from the onset of flow, a linear velocity profile is initially observed, with a slope that is consistent with the nominally applied shear rate. The linear profile persists until the maximum in the $N_{1}^{p}$ overshoot occurs, at which time the velocity profile localizes into separate regions of differing slopes, each associated with a distinct value of local Wi and representing shear bands induced by the microstructural deformation of the constituent macromolecules. The polymer concentration is also essentially uniform and homogeneous at short times; however, concomitantly with the localization of the velocity profile, a fluctuation in the polymer concentration spanning the *y*-direction is observed, likely induced by the relative migration of polymer chain particles as they localize into distinct shear bands through configurational rearrangements. The shear-banded structure and manifestation of concentration fluctuations persists throughout the strain period where $N_{1}^{p}$ is proceeding toward a steady state, exhibiting different values within the apparent shear bands. The shear bands and concentration fluctuations eventually dissipate and steady-state uniform velocity and concentration profiles are established at a single value of Wi, as consistent with the value of shear rate applied through motion of the cell boundaries. This gradual relaxation process of the velocity and concentration profiles can require up to 200 strain units to complete.

Under startup of shear flow at Wi=15 as depicted in Figure 9a, where $\dot{\gamma }<\tau_{R}^{-1}$, the velocity profile is linear and the concentration profile approximately constant at low strain values; however, at γ>5.5, two distinct localized and essentially linear velocity profiles emerge, which induces a rather significant concentration fluctuation of about ±8% across the simulation cell. A microstructural analysis of the polymer molecules occupying each band allows for calculation of $S_{xy}$ for each band, which provides a rather sensitive mechanism for estimating the shear rate within each band using the fact that $S_{xy}$ changes substantially with Wi (see Figure 3a), even within the stress plateau region where $\sigma_{xy}^{p}$ exhibits only nominal growth with increasing Wi. Within the slow band, $S_{xy}\approx 0.05$ corresponding to Wi≈8, whereas in the fast band, $S_{xy}\approx 0.032$ implying Wi≈100. Furthermore, the computed concentration profiles indicate a flow-induced accumulation of polymer chain particles in the slow shear band and a depletion in the fast band. This behavior indicates a flow-induced diffusion of polymer chain particles from regions of low viscosity to those of high viscosity. Moreover, a mild reverse flow (RF) [11], where the velocity profile has a negative slope implying flow in the opposite direction to the imposed kinematics, is observed upon incipient shear band formation at γ≈6, which persists for a short period of time before the slope turns positive again at γ≈18 (this RF phenomenon will be discussed in greater detail below).

Figure 9b displays the startup flow profile at Wi=150 ($\dot{\gamma }>\tau_{R}^{-1}$). Despite the similarity in the developed strain-localized flow structure, tracking the concentration profile reveals an interesting phenomenon: in contrast to the previous case of Wi=15, polymer particles congregate at the fast band at the onset of shear banding (at γ≈8), with a peak concentration of about 20% above the bulk concentration, whereas the polymer concentration in the slow band decreases by up to 5% (see the inset at γ=8 in Figure 9b). These data indicate that the initial stretching and alignment of chains in the fast band results in an increase in the particle concentration in regions of high shear rate. Evidently, the extension of the polymer molecules in the fast band drags particles from adjacent slow bands, lowering the polymer concentration in the slow bands. Meanwhile, there is again evidence of reverse flow at γ=8–18, where the local velocity profile assumes a negative slope. During the RF period, the recoil/retraction of chains within the slow band (see the discussion below) induces a diffusion of polymer particles from the fast band (lower viscosity, higher Wi) to the slow band (higher viscosity, lower Wi), normalizing the concentration distribution within the simulation cell by the time the shear bands dissipate as the system approaches steady-state behavior. Microstructural analysis for each band reveals that the ensemble-averaged $S_{xy}$ of segments in the slow band is approximately 0.056, corresponding to Wi≈4, whereas $S_{xy}=0.02$ in the fast band, corresponding to Wi≈750. The shear bands dissipate after approximately 200 strain units, leading to a uniform linear velocity profile corresponding to the imposed strain rate of Wi=150.

**Figure 10:**
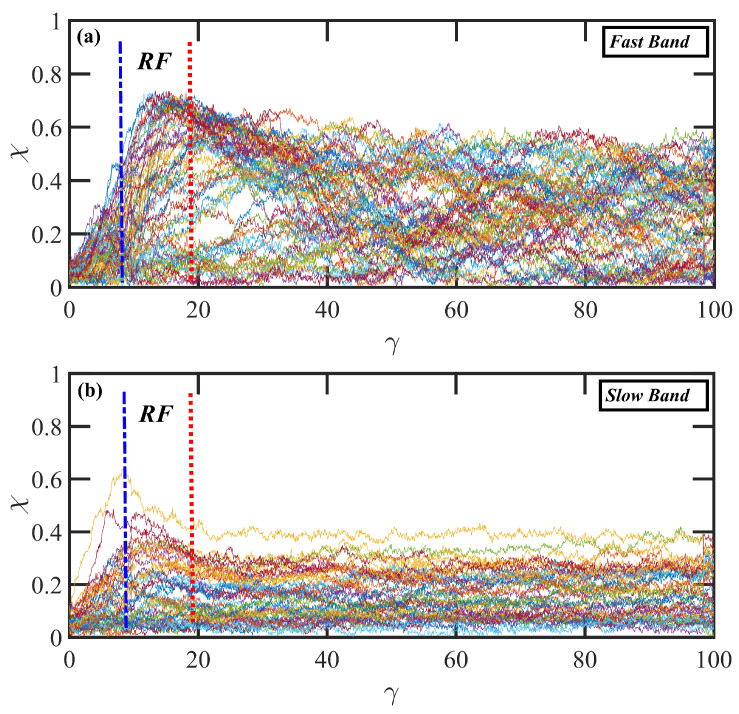
Temporal evolution after startup of shear flow at Wi=150 of individual chain relative extension χ in the fast (**a**) and slow (**b**) shear bands for the PE solution N-1000 in hexadecane (see Figure 9b), where individual chains are denoted by random colors. The panels illustrate the dynamics of chains with their centers of mass located within layers of thickness y/H=0.2: (**a**) the fast band centered at y/H=0.8 and (**b**) the slow band centered at y/H=0.3. Note that the strain region where reverse flow was observed is identified by the dashed blue and dotted red vertical lines within each panel.

To understand the mechanism leading to reverse flow at low strain values, individual chain conformations are tracked and plotted as fractional extension χ vs. γ in Figure 10 for startup of shear flow of the N-1000 hexadecane solution at Wi=150. In this figure, individual molecules are assigned random colors and segregated into slow and fast bands according to their centers of mass at the initiation of shear banding (γ=8). The strain region wherein RF is observed is denoted in this figure by the blue and red vertical lines; i.e., 8<γ<18. The initial behavior of the molecules upon startup of flow is very similar to that observed in Figure 8c,d; i.e., the molecules in both bands stretch out, with those in the fast band attaining higher values of χ than those occupying the slow band. However, in cases where RF occurs, the molecules occupying the slow band experience a recoil after band formation, which occurs throughout the period wherein RF is observed. At the same time, molecules occupying the fast band continue to extend. This behavior is entirely consistent with a similar RF phenomenon observed in DPD simulations of PE melts [11]. At γ>18, the molecules in the fast band begin to experience tumbling cycles, whereas those in the slow band assume pseudosteady-state flow-aligning configurations that persist until an eventual steady state is attained at γ>200 where the distinct shear bands have dissipated and a uniform, linear velocity profile is established throughout the simulation cell.

Startup of shear flow at Wi=1500 of the N-1000 hexadecane solution is displayed similarly to the above cases in Figure 9c. The maximum in the shear stress overshoot occurs at γ=9, with the maximum in $N_{1}^{p}$ (not shown) occurring at γ=12. Trends in the velocity and concentration profiles are analogous to those at Wi=150, as discussed above. In this case, however, the significantly higher applied shear rate generates a rather chaotic flow situation, where the shear bands are constantly evolving up to γ≈50, with large concentration fluctuations (of up to 40%) apparent during the steep decline in both shear stress and first normal stress difference. This appears to be a consequence of the frequent cyclical retraction/extension excursions of the polymer molecules in the plane of shear, as primarily located within the fast band, although at this Wi, both bands are located at $\dot{\gamma }>\tau_{R}^{-1}$, where significant rotational chain dynamics are evident. These observations are consistent with the values of $S_{xy}$ as determined from the shear bands, with a value of 0.01 in the fast band, corresponding to Wi≈5000, and 0.025 in the slow band, corresponding to Wi≈300. At γ>50, the velocity and concentration profiles gradually merge together into a homogeneous flow, with the velocity profile assuming a linear form with a slope proportional to the applied Wi after approximately 200 strain units. No reverse flow is observed at this Wi, which is likely due to the fact that the slow band in this case has an effective $\dot{\gamma }>\tau_{R}^{-1}$, and hence no recoil occurs of the polymer molecules that inhabit it.

Taken all together, the above data indicate that when the applied $\dot{\gamma }<\tau_{R}^{-1}$, the transient shear bands generated at startup tend to consist of a fast band with $\dot{\gamma }>\tau_{R}^{-1}$ and a slow band with $\dot{\gamma }<\tau_{R}^{-1}$. In these cases, a reverse flow might be observed at the initiation of shear banding. In cases where the applied $\dot{\gamma }\gg \tau_{R}^{-1}$, the fast band exhibits an effective $\dot{\gamma }$ much higher than the applied value, and the slow band experiences a lower value, even though $\dot{\gamma }>\tau_{R}^{-1}$, with no evidence of RF. It must be recognized, however, that the cases examined above are not deterministic. In an actual experiment, the physical boundaries can impose a regularity on the system that is not present in a simulation employing periodic boundary conditions. Because of the stochastic element of the simulations, and the breadth of the shear-stress plateau, rerunning simulations, even with the same initial system configuration, is likely to lead to different startup behavior with fast and slow shear bands arising at different locations within the simulation cell and with different effective Wi than those described above. Hence, a spectrum of possible shear-banded structures can exist, and each occasion of startup could result in a different evolution of the velocity and concentration profiles as the system homes in on its eventual steady state from a highly localized microstructural environment that is randomly generated upon inception of flow. An analogous type of behavior was observed recently in startup shear simulations of a similar PE melt [11], although in that case there was only a finite number (3) of possible flow state points due to the nonmonotonicity of the shear stress vs. shear rate flow curve.

**Figure 11:**
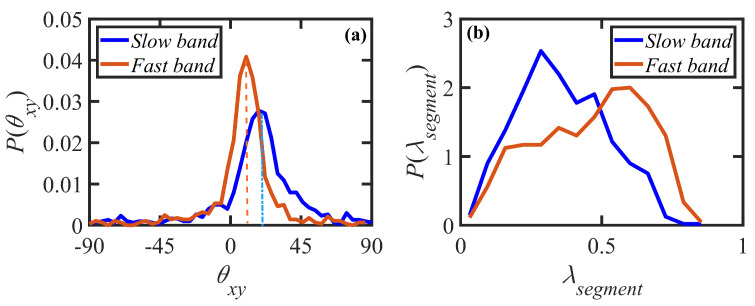
(**a**) The probability distribution function of segmental orientation angle, $P(\theta_{xy})$, and (**b**) the probability distribution function of segmental stretching parameter, $P(\lambda_{segment})$, associated with the segments within the fast (red lines) and slow (blue lines) bands at γ=8 for the PE solution N-1000 in hexadecane undergoing startup shear flow at Wi=150.

Examination of the probability distributions of $\theta_{xy}$ and $\lambda_{segment}$ are displayed in Figure 11 for startup of shear flow of the N-1000 hexadecane solution at Wi=150 at the applied strain γ=8, which is approximately the time of initiation of the shear-banded structure. Here, the segmental stretch is defined as $\lambda_{segment}=\left|R_{seg}\right|/R_{seg_{\max}}$, where $|R_{seg}|$ and $R_{seg_{max}}$ are the magnitude of the segment end-to-end vector and the maximum length of a segment, respectively. Panel (a) demonstrates clearly that the initial stage of shear banding is accompanied by the generation of distinctly different orientation angle distributions within the fast and slow bands, with peak values of 10° in the fast band and 18° in the slow band. Hence, polymer molecules within the fast band are decidedly more oriented along the flow direction than those within the slow band. Panel (b) indicates that segmental stretching is also vastly different between the two shear bands, with the fast band being composed of more highly stretched chains with a distribution peak at 0.60, whereas the slow band is composed of more coiled chains with a peak at 0.28. These observations offer a plausible explanation for the development of the shear bands within certain layers of the simulation cell where localized differences in molecular configuration and entanglement density (see below) that arise from the stochastic nature of the chain dynamics lead to temporary band formation at approximately the same value of shear stress but with distinct $N_{1}^{p}$ values, as observed in Figure 8b.

**Figure 12:**
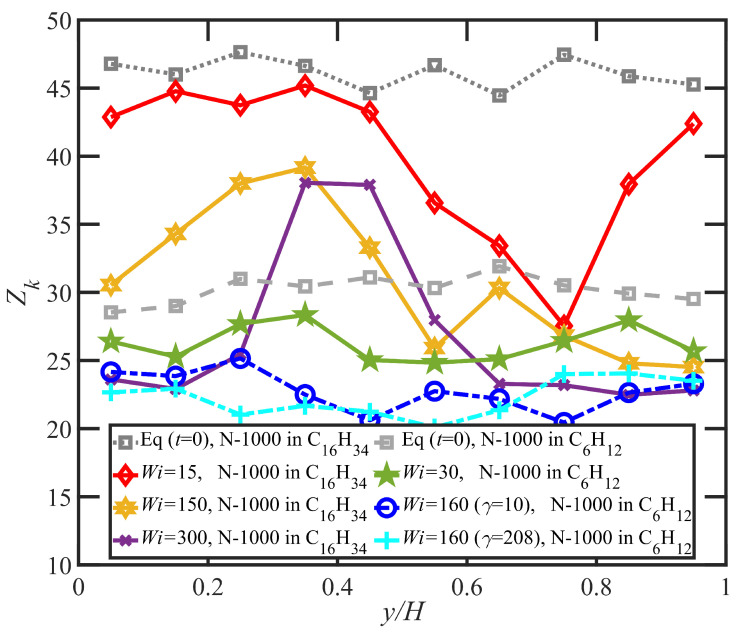
The number of kink entanglements per chain averaged within respective layers of the simulation cell spanning the *y*-direction as calculated over the lifetime of the shear bands. Low values of $Z_{k}$ at each Wi indicate layers associated with the fast band, whereas high values correspond to layers within the slow band.

The analysis of the polymer network of Figure 12 demonstrates the difference between the entanglement network within the two distinct flow bands, where the average entanglement kink number per molecule is plotted over ten equally sized layers of the simulation cell in the gradient direction at equilibrium and various Wi for both the hexadecane and benzene solutions. For PE N-1000 in hexadecane, the distribution of $Z_{k}$ at equilibrium (black square data) is uniform throughout the various layers. At nonzero values of Wi, however, layers associated with the fast band have significantly fewer entanglement kinks per chain than layers comprising the slow band. As the applied Wi increases, $Z_{k}$ decreases in both bands, as also evident from Figure 4 for the bulk average value. Indeed, the spatial regions of the simulation cell within which the respective bands are generated is likely associated with inhomogeneities in the entanglement network that begin to develop spontaneously upon inception of flow and attain a critical decomposition point near the maximum of the $N_{1}^{p}$ vs. γ curve.

**Figure 13:**
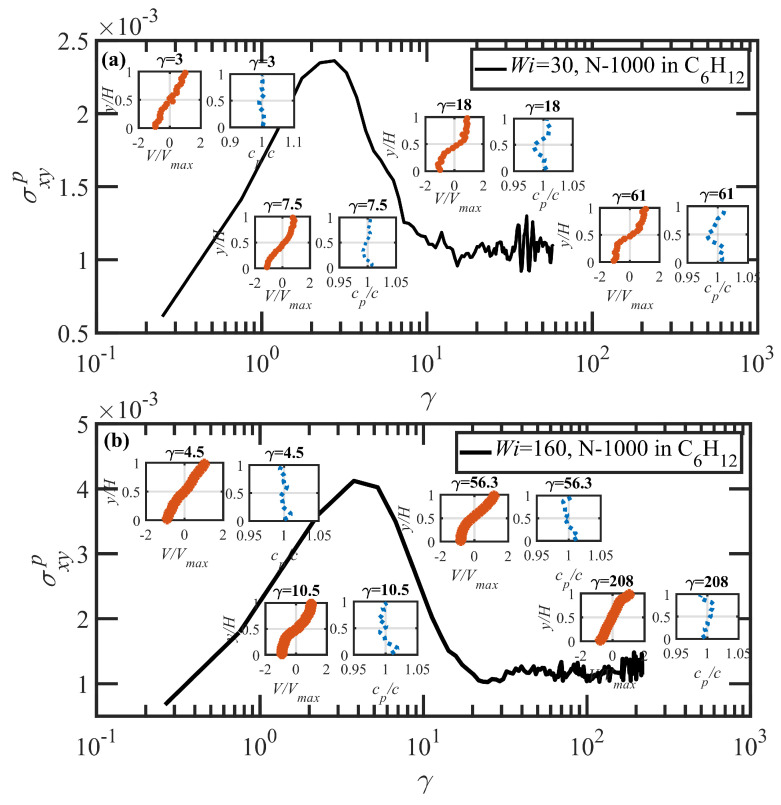

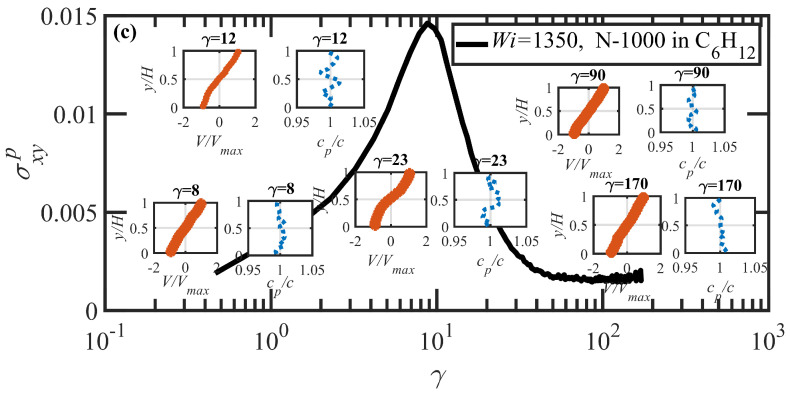
The temporal evolution of shear stress vs. strain for PE solution N-1000 in benzene undergoing startup shear flow at (**a**) Wi=30, (**b**) Wi=160, and (**c**) Wi=1350. The insets display the scaled instantaneous velocity profiles, $V/V_{\max}$, and scaled instantaneous polymer concentration profiles, $c_{p}/c$, spanning the scaled simulation cell dimension in the *y*-direction (y⁄H) at specific values of applied strain.

The time evolution under startup of shear flow for the PE N-1000 in benzene solution at Wi=30, 160, and 1350 is displayed in Figure 13a–c, respectively. In general, the exhibition of shear banding for the N-1000 benzene solution is very similar to that discussed earlier for the hexadecane solution. Shear bands begin to form near the maximum in the overshoot of $N_{1}^{p}$ (γ≈7,9,12) for the three values of Wi noted above, which occurs roughly 3–5 strain units after the maximum in the overshoot of $\sigma_{xy}^{p}$ (γ≈3,5,9). Prior to shear-band formation, the velocity and polymer concentration profiles are uniform and linear throughout the simulation cell, and the same observations are evident once the final steady-state condition is attained after several hundred strain units have transpired. At low Wi (30 and 160), the slow bands again show a relative increase in polymer concentration, whereas the fast bands indicate a depletion. This trend reverses itself at high applied Wi, where an accumulation is observed in the fast bands relative to the slow bands, as also observed in the hexadecane solution. In general, concentration fluctuations in the benzene solution are much less intense than in the hexadecane solution, rarely exceeding more than ±3%; this is likely due to the much faster dynamics of the small molecule benzene solvent, which can remain more evenly distributed throughout the simulation cell during the course of shear startup. No reverse flow is observed in the benzene solution startup simulations, which could be due to the significantly lower number of entanglements in benzene at low Wi, as evident in Figure 4 and Figure 12. The entanglements thus appear to be the primary driver of the recoil in the slow bands (see Figure 10b) that produces the RF phenomenon.

At Wi=30, $S_{xy}$ in the fast band averages to 0.04 and 0.053 in the slow band. These values correspond to effective Wi in the fast and slow bands of approximately 60 and 7, respectively, similarly to the hexadecane solution. Both values are essentially at or below the critical value of $\tau_{R}^{-1}$. At Wi=160, $S_{xy}=0.025,\;0.050$ in the fast and slow bands, indicating Wi in the fast and slow bands of 500 and 10. At Wi=1350, $S_{xy}$ has a fairly small value (≈0.02), which is of the same order of magnitude as the fluctuation in $S_{xy}$ at these high values of Wi where the molecules are experiencing frequent molecular tumbling cycles. Because of this, it is very difficult to estimate the effective Wi of the fast and slow bands; however, both are clearly at $\dot{\gamma }\gg \tau_{R}^{-1}$.

## 4. Conclusions

Transient and steady-state dynamic responses of entangled linear polyethylene solutions subject to the startup of shear flow were investigated via a suite of high-fidelity nonequilibrium dissipative particle dynamics simulations. The coarse-grained DPD model [42] was developed to replicate entangled macromolecules consisting of 1000-bead chains corresponding to monodisperse linear PE liquids of $C_{3000}H_{6002}$ dissolved in hexadecane and benzene solvents. Below is a list of key conclusions from this study.

- Both N-1000 PE hexadecane and benzene solutions exhibited a monotonic steady-state shear stress profile when plotted versus applied strain rate with a very broad stress plateau at intermediate Wi.
- Under startup of shear flow, both solutions initially exhibited uniform, linear uniform velocity profiles and homogeneous polymer concentration. However, at an applied strain approximately that at which a maximum occurred in the overshoot of the first normal stress difference, transient shear bands developed within the simulation cell for a period of time ranging up to several hundred strain units. During this strain period, the velocity profile across the cell in the gradient direction was not uniform, with two or more local zones of relatively low and high strain rates. At high strains, the shear bands dissipated and the system attained steady-state behavior, with a uniform, linear velocity profile across the simulation cell and a homogeneous concentration. This transient shear banding was observed throughout the applied Wi corresponding to the shear stress plateau.
- Regardless of whether or not shear bands occurred, the shear stress was homogeneous throughout the simulation cell, as verified using various sublayers of the simulation cell in the gradient direction. However, during the lifetime of the shear bands, the first normal stress difference was significantly different between the slow and fast bands, with a higher value in the fast bands where the individual molecules were more highly extended.
- When the effective $\dot{\gamma }$ of the slow band is less than $\tau_{R}^{-1}$, the polymer concentration is inhomogeneous with an accumulation of chain particles in the slow band and a depletion in the fast band. However, when the effective $\dot{\gamma }$ of the slow band is larger than $\tau_{R}^{-1}$, the opposite trend is observed, with chain particles preferentially migrating into the fast band and depleting the slow band. These results concerning nonuniform concentration profiles in shear flow of polymer solutions are consistent with those of prior experimental work [35,36], although evidence presented herein implies that shear bands develop solely due to the polymer chain dynamics, rather than necessarily being prerequisite to a nonuniform concentration profile, as suggested by Burroughs et al. The nonuniform concentration profiles generated in the present simulation work appear to be merely the result of different chain dynamics arising in the fast and slow bands.
- Variations in local orientation and stretching of the tube network segments and the commensurate destruction of the entanglement network appear to be the primary driving mechanisms of the transient shear banding. Molecules within the fast and slow bands experience different topological environments, as induced by the stochastic nature of the flow. Molecules within the fast bands have higher fractional extension and experience quasiperiodic extension/retraction cycles, whereas those in the slow band have lower fractional extension and tend to exhibit a flow-aligning behavior during the lifetime of the shear bands.
- In some instances, a mild reverse flow (i.e., a negative local velocity) was observed after the onset of shear banding. This phenomenon resulted from elastic recoil associated with the molecules within the slow band upon inception of shear banding as the entanglement network fractured along the interface between the bands. The entanglement kink number within the slow band remained significantly higher than the concomitant number in the fast band. Reverse flow was only observed in the hexadecane solution and not the benzene solution. This might be indicative of the much lower number of entanglement kinks in the benzene solution at low applied Wi.
- It is highly likely that a multitude of possible shear bands could be observed if further simulations had been possible, due to the stochastic nature of the DPD algorithm (and experiments). At the stress plateau, the stress values, although strictly monotonic, are increasing at such a low slope that the system dynamics could temporarily become trapped in any number of comparable stress states at any particular time, resulting in a chaotic instability that gradually evolves into a steady-state uniform velocity profile at high strain values. This type of instability could conceivably generate a spectrum of shear bands of differing strain rate within which $\dot{\gamma }$ is evolving in time.
- The underlying mechanism of shear band formation in polymer melts and solutions is essentially the same, being driven by flow-induced disentanglement and localized, individual chain dynamics that are stochastic by nature. In dense melts, however, the concentration inhomogeneity associated with shear banding observed in solutions is effectively negligible. Shear bands develop at shear stress values that possess a multiplicity of compatible shear rates. Different shear-rate zones correspond to varying configurational dynamics of the constituent polymer molecules. Because of the broad spectrum of available molecular configurations of the chains under flow, it is possible that multiple configurationally dynamic states can be associated with a single value of imposed shear stress. Hence, the primary physical mechanism underlying shear banding in both polymer solutions and melts is evidently the same.

Overall, step-strain shear flow simulations revealed the development of spatial inhomogeneities and dynamic instabilities in entangled polymeric solutions. Specifically, it was shown that flow perturbations arose soon after the occurrence of a large normal stress overshoot and that banded flow structures stemmed from inhomogeneous chain segmental orientation and entanglement density in the flow gradient direction. Hence, the mechanism for incipient shear banding in entangled polymeric solutions is identical to that of entangled polymeric melts. This universal mechanism negates the necessity of nonuniform polymer concentration in the flow gradient direction for the formation of incipient banded flow structure in entangled polymeric solutions.

The impact of understanding the mechanism of shear banding in polymer solutions (discussed herein) and melts (investigated in a prior simulation study [11]) could be profound and help to explain any number of previously poorly understood phenomena observed in viscoelastic fluid mechanics, such as extrudate distortion in polymer melt extrusion [1,2], which have been traditionally focused on the failure of the no-slip boundary condition assumed in continuum mechanics theory. Indeed, as Denn has suggested [2], “Shear bands (discontinuities in the velocity gradient) will exist in the flow field above the transition, and these could be indistinguishable from slip in a macroscopic experiment; indeed, shear banding is a possible mechanism for the formation of a lubricated low-viscosity region adjacent to the wall”. Given that extrudate distortion is observed in the same shear stress region as shear banding, it is a remarkably insightful and probable suggestion to consider such flow instabilities derived from shear banding as a contributor to extrudate distortion. In light of results from recent experiments and simulation studies, it might be worthwhile to revisit some of these prior investigations into flow instabilities from a more fully developed perspective.

## Figures and Tables

**Table 1:** DPD simulation parameters used in this work. These parameters were chosen systematically by comparisons with equilibrium and nonequilibrium molecular dynamics simulation data of atomistic polyethylene liquids [42].

Parameter	Value	Units
*a*	200	$k_{B}T/r_{c_{a}}$
$\gamma^{D}$	4.5	${\left(mk_{B}T/r_{c_{a}}^{2}\right)}^{1/2}$
σ	3.0	${\left(m{\left(k_{B}T\right)}^{3}/r_{c_{a}}^{2}\right)}^{1/4}$
Δt	0.012$\tau_{DPD}$	${\left(mr_{c_{a}}^{2}/k_{B}T\right)}^{1/2}$
$k_{bend}$	2.38 (N-1000) and 2.0 ($N_{H}$-6)	$k_{B}T$
$k_{spr}$	400	$k_{B}T/r_{c_{a}}^{2}$

**Table 2:** Dimensions of the simulation cells for each melt. $L_{x}$, $L_{y}$, and $L_{x}$ are displayed as multiples of the equilibrium value of $R_{g}$ ($\equiv {\langle R_{g}^{2}\rangle }^{0.5}$) and also in dimensionless $r_{c_{a}}$ units (displayed within parentheses). The total number of DPD particles used in the respective simulations is also tabulated.

Chain Length	$L_{x}$	$L_{y}$	$L_{z}$	$N_{p}$	$N_{\mathrm{total}}$
N-1000 in hexadecane	16$R_{g}$ (310)	4$R_{g}$ (84)	1.5$R_{g}$ (31)	370,000	791,380
N-1000 in benzene	16$R_{g}$ (310)	4$R_{g}$ (84)	1.5$R_{g}$ (31)	240,000	590,000

**Table 3:** Equilibrium properties of the simulated PE solutions. Entries are displayed in DPD units.

DPD Chain	ϕ	$c/c^{*}$	${\langle R^{2}\rangle }^{0.5}$	${\langle R_{g}^{2}\rangle }^{0.5}$	$\tau_{d}$	$\tau_{R}$	$Z_{k}$	*Z*	$N_{K}$
N-1000 in hexadecane	0.46	20	62	24.5	$6.4\times 10^{7}$	$8.53\times 10^{5}$	46	25	234
N-1000 in benzene	0.3	15	61.33	25.2	$5.0\times 10^{7}$	$9.8\times 10^{5}$	30	17	236

## Data Availability

Data generated in the simulations reported in this paper are available from the authors upon reasonable request.
